# Does Long-Term Enrollment in Day-Care Maintain or Increase Early Developmental Gains—Findings from an Intervention Study in Rural Bangladesh

**DOI:** 10.3390/children9070929

**Published:** 2022-06-21

**Authors:** Priyanka Agrawal, Divya Nair, Shumona Sharmin Salam, Md Irteja Islam, Jena Derakhshani Hamadani, Olakunle Alonge

**Affiliations:** 1 Department of International Health, Johns Hopkins Bloomberg School of Public Health, Baltimore, MD 21205, USA; divyanair.dna@gmail.com (D.N.); oalonge1@jhu.edu (O.A.); 2 Department of Oncology & Metabolism, The University of Sheffield, Jessop Wing, Tree Root Walk, Sheffield S10 2SF, UK; shumona.salam@sheffield.ac.uk; 3 Faculty of Medicine and Health, Sydney School of Public Health, The University of Sydney, Edward Ford Building, Camperdown, Sydney, NSW 2006, Australia; m.i.islam@sydney.edu.au; 4 Maternal and Child Health Division, International Centre for Diarrheal Disease Research, Bangladesh (icddr,b), GPO Box 128, Dhaka 1000, Bangladesh; jena@icddrb.org

**Keywords:** cognitive development, early childhood development, community day-cares, children, rural Bangladesh

## Abstract

Objective: Community day-care centers (or crèches) are gaining popularity; access to these centers can reduce cognitive gaps. This paper describes the sustained impact of enrollment in day-cares on cognitive gains. Methods: As part of a larger study, a census of all children was conducted in 2012–2013 to identify children between 9 and 17 months of age in rural Bangladesh. A sub-sample of children (*n* = ~1000) were assigned to receive either a day-care or playpen. Children from two sub-districts were randomly selected and assessed at 9–17 months of age for cognitive and behavioral domains using the Ages and Stages Questionnaire-III. The same children were then followed-up with after one year to see if the scores obtained by the children in the day-care intervention were different from those enrolled in the playpen intervention using a difference-in-difference estimator. Results: Children enrolled in the day-care intervention performed better (in communication, gross-motor, personal-social, and problem-solving domains) than children enrolled in the playpens when followed up with after a one-year period. Total scores were 0.31 (95% CI 0.141–0.472) higher (*p* value < 0.001) among children in the day-cares. Family care indicators as well as the child’s and mother’s weight were significantly associated with sustained and increased cognitive gains. Conclusion and relevance: The cognitive and psychosocial improvements seen with short-term exposure to structured ECD programs (day-care) were observed to be sustained over time with continued exposure. Home stimulation and parental involvement add to the long-term benefits of ECD.

## 1. Introduction

Global evidence around the benefits of early childhood development (ECD) interventions for developmental and cognitive stimulation of young children has increased in recent years. ECD interventions prioritize age-appropriate and nurturing care that is focused on child behaviors, attitudes, stimulation, responsiveness, and safety, and these strategies have overall improvements in quality of life for children [1,2]. However, 43% of children under 5 years of age in low- and middle-income countries (LMICs) miss out on these important strategies and are still at risk of not achieving their developmental milestones [3,4]. This can be attributed to a combination of issues, ranging from widespread poverty, infectious diseases, malnutrition, poor sanitation and hygiene, and a lack of household resources for cognitive stimulation and learning opportunities within LMICs [5].

In many LMICs, parental counselling during home visits or at health centers used to be the main approach for delivering ECD interventions, with the aim of stimulating cognitive development in children [6,7]. However, out-of-home day-cares have now become more popular in recognition of the benefits of ECD interventions; primary caregivers (mostly mothers) in LMICs are seeking employment and income outside of the home setting. Despite this popularity, day-cares have low systematic coverage and face significant systematic barriers such as poorly maintained spaces as well as a lack of age-appropriate structured curriculum and trained caregivers. Hence, there is mixed evidence regarding the effectiveness of day-cares in stimulating the cognitive developments of children in LMICs [8,9].

The Saving of Lives from Drowning (SOLID) project, a multi-year research study, was implemented to study the large-scale effectiveness and cost-effectiveness of two drowning prevention interventions—day-cares and playpens—among children under five years of age. Although the project was primarily implemented to address drowning prevention among children aged 9–47 months, the day-cares were designed with the intention of providing cognitive stimulation to children attending the day-cares [10,11]. The day-care (also known as ‘anchal’ in the local language, Bangla) curriculum included activities to build social skills, personal hygiene habits, nutrition, and learning (including age-appropriate mathematics, language skills, geography, and local flora and fauna lessons) supplemented with age-appropriate toys and reading materials in the local Bangla language [10]. Women from the communities were trained to run the day-cares as anchal mothers and assistants, 9 a.m.–1 p.m., six days a week. The day-cares were tuition-free and had an average of 20 to 22 children each. Union-, village-, and district-level government agencies were involved in decision making and providing support to the women to facilitate ownership among communities.

The implementation of phase one of the SOLID project over a long period of time (2013–2016) provided an opportunity to evaluate the cognitive and psychosocial benefits of enrolling children under the age of 5 in day-cares. An initial assessment showed that exposure to early childhood education was associated with higher cognitive gains among the children enrolled [10]. In this paper, we describe the findings from a one-year follow-up of children enrolled in day-cares and establish whether the observed cognitive gains were maintained or changed over time. We further explore the effect of prolonged exposure to day-cares on their psychosocial outcomes. We hope this paper will contribute to the evidence on the cumulative gains of prolonged enrollment in day-cares in resource-limited settings.

## 2. Methods

The SOLID project was set in seven rural sub-districts of Bangladesh (Raiganj, Sherpur, Manohardi, Matlab South, Matlab North, Daudkandi, and Chandpur Sadar) and targeted all children aged 9–47 months residing in the seven rural sub-districts. The SOLID project established day-cares for drowning prevention across the seven rural sub-districts and also included a playpen intervention distributed to households with eligible children on a ‘‘first come first serve’’ basis [11]. Community sensitization efforts were simultaneously made to generate buy-in and encourage all households in the seven sub-districts to participate in the study.

**Figure 1:**
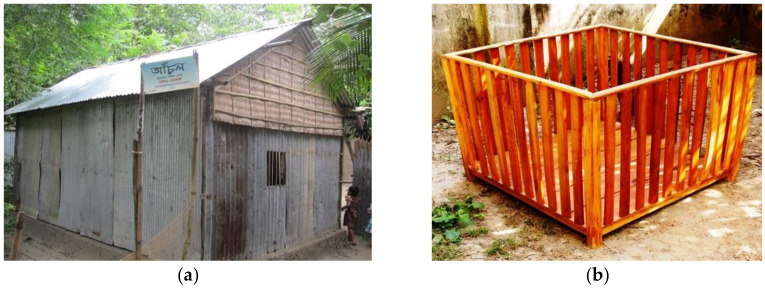
SOLID study interventions: (**a**) day-care intervention; (**b**) wooden playpen.

Unlike day-cares, a playpen is a home-based apparatus that the caregivers were encouraged to keep their children in whenever they were busy with household chores. The playpen was not expected to confer cognitive stimulation benefits (Figure 1). Hence, a sub-study was nested within the larger project to assess the cognitive outcomes of the subset of children aged 9–17 months exposed to the day-cares. The sub-study was designed to include children selected from two (Matlab South and Matlab North) of the 7 sub-districts from the larger project, and these two districts were purposefully selected because of their large population.

**Figure 2:**
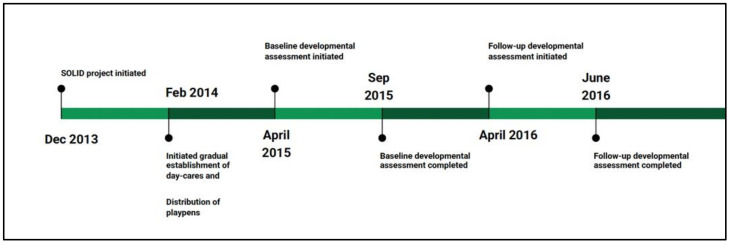
Timeline for data collection of developmental assessment study.

The two sub-districts were divided into two areas, with the villages in those sub-districts being randomly allocated into Area 1 or Area 2. In Area 1, playpens were initially distributed to a limited number of children while the day-cares were gradually established. Playpens were also distributed on a first-come, first-served basis to children who were eligible to receive the playpen. In Area 2, day-cares were gradually established over the entire study period while playpens were distributed on a ‘first-come first-serve’ basis after the initial period. Children in both areas had the choice of participating in either or both interventions. For the sub-study, villages under Area 1 and 2 were randomly selected to include the children enrolled in playpen-only group (in Area 1) and in the day-care-only group (in Area 2). Children enrolled in both the playpen and day-care interventions across Areas 1 and 2 were not included in the sub-study. At the time of the initial developmental assessment (considered the baseline assessment), i.e., between April and September 2015, 30 children aged 9–17 months were selected from each village until a total of 600 children was selected from each area, for a total of ~1200 children for the sub-study [10]. A follow-up assessment was conducted for the same set of ~1200 children approximately 12 months after the first assessment between April and June 2016 (Figure 2).

Based on the baseline assessment, it had been established that children exposed to the day-care intervention (selected from Area 2) had higher developmental and cognitive gains than children in the playpen intervention (selected from Area 1) [10]. In this paper, we examined whether those gains were preserved and/or maintained by the time of the follow-up assessment.

The Ages and Stages Questionnaire (ASQ-3), a developmental screening tool to study children under 5 years of age, was implemented to measure cognitive and psychosocial outcomes in both the baseline and follow-up assessments [12]. The ASQ-3, which was translated into Bangla and modified to suit the local context, assesses five domains of ECD—gross motor, fine motor, communications, problem-solving, and socioemotional development [9]. Each domain consists of a block of 12 questions, and parents (mainly mothers) or a caregiver are the main respondents. Some questions directly test a child’s performance on certain activities (Table 1). 

The baseline assessment was conducted on children aged 9–17 months, and the follow-up assessment was conducted after one year on the same children, who were then aged 21–36 months. Control variables that may explain the ECD domains from the ASQ-3 were collected using a family questionnaire. Variables included the gender, age, head circumference, and weight-for-age z scores of the children at the time of the interview, family care indicators (FCIs), the household asset index, the mother’s weight, and disability. The FCIs were derived from the Home Observations for Measurement of the Environment (HOME) questionnaire, which includes a standard set of questions around items (such as items used to play music, color, pretend play, movement, shapes) that a child interacts with in the home environment [13,14]. The indicator serves as a proxy measure of home-based stimulation for children under 5 years of age. Each question had a yes/no response, and scores were added to a total score of seven. The household asset index was calculated as a measure of the SES using a principal component analysis of the data for household assets; land; electricity; source of drinking water; toilet facilities; electronic; vehicles; main material of the wall, roof, and floor; and bank accounts. For each child, it took on average 30 to 35 min to complete the ASQ, 10–15 min to complete the FCIs, and 10 min for anthropometry, for a total of 50–55 min.

Unadjusted bivariate associations between the test scores for children in day-cares (and playpen) and their background characteristics (control variables) were tested using the *t*-test, chi-square, and ordinary least square regressions.

For each round, the outcome scores from each ASQ-3 domain (gross motor, fine motor, communications, problem-solving and socioemotional development) were standardized as age-adjusted z-scores. We hypothesized that the developmental gains observed during the first round of data collection among children under the day-care intervention are at least sustained, if not increased over time. We further hypothesized that a similar association will not be observed or that the magnitude of the association will be significantly smaller for those under the playpen intervention. As the length of exposure to either intervention at the follow-up assessment was the same for all children (~12 months), an ordinary least square regression model was used to regress the difference in the age-adjusted z-scores between round 2 and round 1, adjusting for all other control variables. To account for the cluster sampling design, site-specific clustered standard errors (at the village level) and fixed-effect estimates were used to improve estimates.

Ethical approval (IRB 00004746) for this study was obtained from the institutional review boards of the Johns Hopkins Bloomberg School of Public Health and the International Centre for Diarrhoeal Disease and Research, Bangladesh, on 20 November 2014.

## 3. Results

On following up with the same 1018 children who were included in the final analytical sample during the baseline ASQ assessment, data for 114 children who were part of the follow-up data collection were incomplete and thus dropped. The final analytical sample resulted in a total of 904 children (21 to 36 months of age at the time of follow-up data collection) who had complete data sets for both the baseline and follow-up ASQ assessment. Of these, 466 children had been enrolled in the day-care intervention, and 438 children had been enrolled in the playpen intervention at baseline. By follow-up, the children in the playpen and day-care interventions had received an average of 443.35 days and 389.93 days of intervention exposure, respectively (a difference of approximately 2 months, *p* value < 0.001), like what was seen in round one (playpen—115.37 days; day-care—53.42 days).

Without adjusting for control variables, children enrolled in the day-cares had lower scores for all psychosocial domains, with significantly different and lower scores for the gross motor and personal/social domains (Table 2). Lower body weight was significantly associated with children enrolled in the day-care intervention. Family care indicators were significantly different and lower among children enrolled in the day-care intervention (*p* value < 0.001).

After adjusting for the control variables, children who were exposed to the day-care intervention at baseline and who continued to be enrolled in the day-cares up until the follow-up assessment had higher total and domain specific scores, except for fine motor scores, than children who had continued exposure to the playpens in both rounds one and two (Table 3). Longer exposure time were associated with a unit increase in the total score by 0.31 (95% CI 0.141–0.472), with an additional day of exposure to the day-care intervention at a p value of less than 0.001. Children with higher z-scores for their weights and maternal weights were associated with better ASQ scores. A higher asset index was associated with lower scores. Longer exposure was associated with better communication (*p* value < 0.01) and problem-solving scores (*p* value < 0.001) as well as total scores (*p* value < 0.01).

To test our hypotheses that children enrolled in day-cares have sustained if not increased gains by round two, a difference-in-difference estimate was calculated. The difference in the z-scores for each ASQ domain at round two versus round one was regressed for all control factors (Table 4). Taking into consideration that the exposure period for all children included in the analysis was ~330 days irrespective of whether they were enrolled in the day-care or playpen intervention, a slight increase was seen in the change in the z-scores across all ASQ domains (except fine motor skills) of the children enrolled in day-cares compared to those enrolled in the playpen intervention when the DID estimator was applied. However, the positive change in the scores was not significantly associated with being enrolled in a day-care.

## 4. Discussion

Children enrolled in the day-care program had better communication, gross motor, personal-social, problem-solving, and total ASQ scores than children enrolled in playpens after an additional year of continuous exposure to both interventions. However, the difference in the difference estimator suggests that the early gains obtained from being exposed to a day-care may be lost over time if early childhood education and stimulation in the form of structured day-cares is not continued [10,15]. There were minor gains in the communication, problem solving, and emotional–social category with enrollment in day-cares; however, the gains were non-significant. These findings highlight the need for continuous exposure to early childhood stimulation to sustain any initial developmental benefit that may have been gained through a day-care program. The brain of a child is actively evolving during different stages of his or her early years, and nurturing care during each of these stages may be important to maintain lifelong benefits. Hence, day-care exposure should not be seen as a single, time-limited activity delivered for a short period of time, but a continuous delivery over a significant period, preferably continuing until the child is enrolled in elementary school. Past studies have shown long-term benefits of pre-school education programs on school performance, social adjustment, and reduced violent tendencies in addition to immediate and short-term gains, as seen with this study [16,17,18].

The need for continuous day-care exposure has financial and planning implications, especially for rural populations such as those included in this study. The private market may not be adequately resourced to support such services despite the obvious benefits. Hence, it is important for the government and public educational agencies who typically cater for school education in such settings to have ownership of the day-care program from the outset.

The day-care curriculum in this study focused on gross motor skills such as dancing and did not cover fine motor activities such as writing, playing with beads, and arts and crafts [19]. The development of fine motor skills at early ages has been positively linked to higher cognitive development and function in adolescents and adults [19]. Activities such as fingerplay and manipulative play improve hand–eye coordination and prepare children for writing and reading [20]. Not only do fine motor activities increase the readiness of children to participate in school activities, there are physical, psychological, and behavioral gains such as independence, confidence, and self-help [21]. There is potential to revise the day-care curriculum to provide holistic growth and development opportunities. This would also entail additional, rigorous training for day-care mothers and relevant learning materials.

Home stimulation measured through the FCIs showed that higher stimulation and engagement at home led to developmental gains [22,23,24]. This is also linked to higher parental and sibling involvement and accelerates the expansion of phonetics and language overall [25]. Poor households showed lower developmental gains, as has been corroborated from earlier studies [3,26]. Developmental delays during early childhood can result in poor academic achievement in school and low paying jobs in adulthood [27,28]. Mothers can be trained in home-based early childhood activities to enhance the cognitive growth of young children [29]. Additionally, the engagement of both parents supplements home engagement activities and is a strong means to meet the stimulation needs of growing children [30]. Policies and programs to make ECD services available to families from poor households are required to target children to shift the resource poor paradigm to an interactive and enriching environment. Children with disabilities who were enrolled in day-cares scored significantly higher on almost all domains of the ASQ at the one-year follow up compared to the first assessment. Some research has associated integrated ECD programs with better outcomes for disabled children. However, multi-pronged programs and mixed views make it difficult to suggest clear and targeted recommendations [31].

There are some limitations to the present study. The Bangla-translated ASQ-3 has not been validated in the context of Bangladesh. The quality of day-cares and the education level of the day-care mothers were not accounted for as these are known evidence-based indicators associated with cognitive and psychosocial development [32,33]. The study required the day-cares to maintain their cleanliness and supplies and were regularly monitored. Women had to have at least higher secondary-level education to be eligible to be day-care mothers. The non-standardized curriculum and requirements of the set up hinder an objective comparison with differently structured ECD programs. Additionally, it is important to note that the project conducted rigorous community sensitization around the benefits of day-cares—mainly as a drowning prevention intervention, but also highlighted their collateral benefits for children. Additionally, the children in the playpen intervention had consistently higher exposure to the intervention; the logistics played out such that playpens were distributed in the communities before the day-cares had been set up to accept children enrolled into the study. It is also important to note that children enrolled in the playpen intervention had higher psychosocial domain scores, head circumference, body weight, and family care indicators at baseline compared to the children enrolled in the day-care intervention. The unequal distribution of the baseline characteristics potentially had an impact on the findings of the study.

Rigorous studies are needed in LMIC settings to establish standard requirements for the maintenance of day-care spaces and the qualifications of day-care caregivers. Longer follow up studies are needed to see the long-term impact of early childhood education programs on school performance in LMIC settings. Similar long-term follow-ups from high income countries have shown substantial cognitive, behavioral, health, and academic benefits from early childhood interventions [34,35]. These studies include evidence-based information to encourage governments to prioritize the inclusion of ECD programs in national education plans.

## 5. Conclusions

ECD programs are important to promote the development of children in LMICs, and these effects are retained in the mid- to long-term period. Early and continuous intervention has benefits later in life, compounding early investments into future gains, thereby contributing to employability and quality of life for the child into adulthood. ECD programs should be rigorously evaluated with regular, short, and long-term follow up in their specific contexts to account for varied implementation quality. Evidence-based policy discussions and political will to uptake and scale up ECD services at the national level are necessary. Importantly, multi-sectoral collaborations from the perspectives of health, nutrition, education, and women’s empowerment will be required to push forward the agenda to reduce inequity and to promote effective care for young and vulnerable populations.

## Figures and Tables

**Table 1:** Exposure characteristics for the sample population.

Metrics	Baseline Assessment	Follow Up Assessment
Timeline	April–September 2015	April to June 2016
Age group	9–17 months	21 to 36 months
Screening tool	Ages and Stages questionnaire	Ages and Stages questionnaire
Average length of exposure at the time of assessment (days)	Playpen: 115.37 Day-care: 53.42	Playpen: 443.35 Day-care: 389.93

**Table 2:** Sample characteristics and differences between children in playpen and day-care interventions on the ages and stages questionnaire in round 2 (426 days post-intervention on average).

Variables (ASQ Domains)		Playpen (*n* = 438)				Day-Care (*n* = 466)				*p* Value
		Mean	SD	Min	Max	Mean	SD	Min	Max	(Difference across Interventions
Raw scores	Communication	89.26	22.07	0	120	86.61	23.31	0	120	0.08
	Gross motor	83.41	23.32	5	120	79.66	23.57	0	120	0.016 *
	Fine motor	76.87	16.93	20	120	75.55	16.75	20	120	0.237
	Personal/Social	81.54	19.12	15	120	78.51	20.4	5	120	0.022 *
	Problem solving	72.12	18.4	10	115	72.66	18.4	0	115	0.653
	TOTAL SCORE	403.21	77.73	50	555	392.98	80.58	35	555	0.053
Z Scores	Communications	0.31	0.65	−2.09	1.34	0.24	0.68	−2.15	1.34	0.119
	Gross motor	0.3	0.71	−2.36	1.73	0.22	0.69	−1.91	1.73	0.062
	Fine motor	0.29	0.59	−1.92	2.41	0.28	0.58	−1.54	2.41	0.586
	Personal/Social	0.31	0.62	−2.14	1.74	0.26	0.63	−1.58	2.14	0.209
	Problem solving	0.26	0.65	−2.11	1.6	0.31	0.61	−1.77	1.80	0.195
	TOTAL SCORE	0.32	0.54	−2.17	1.31	0.28	0.55	−1.69	1.59	0.286
Controls	Sex (male, %)	54.79				49.57				0.116
	Age	25.09	1.93	21	30	25.14	1.99	21	30	0.743
	FCI ^1^	3.61	1.06	1	7	3.38	1.11	0	7	<0.001 ***
	Asset index	0.06	1	−2.72	3.70	−0.02	1.02	−3.56	2.88	0.187
	Head Circumference	46.05	3.03	42	49.9	45.70	1.32	40	51	0.033 *
	Mother’s weight	51.74	10.07	35.1	89.4	49.69	9.17	35	76.8	0.480
	Child’s weight	11.02	1.73	7.9	26.2	10.74	1.41	6.3	17.5	0.004 **
	Total exposure to intervention (days) ^2^	446.66	49.66	160	537	389.72	48.01	159	6490	<0.001 ***
	Disability	8.82	0.48	5	9	8.90	0.3	8	9	0.216

*** *p* value < 0.001, ** *p* value < 0.01, * *p* value < 0.05, two-sided *t*-tests for continuous variables and chi-square tests for categorical variables. ^1^ Family Care Indicator—derived from the Home Observations for Measurement of the Environment (HOME) questionnaire which includes a standard set of questions around items that a child plays with or interacts with in the home environment. ^2^ Calculated as the total number of days enrolled in the intervention from the day of enrollment to second interview.

**Table 3:** Coefficient estimates from multivariable linear regression models, regressing round two ASQ z-scores on individual and household characteristics.

Variables	ASQ Domains, Coefficients (95% Confidence Intervals)					
	Communication	Gross Motor	Fine Motor	Personal/Social	Problem Solving	Total Score
Day-care (Yes)	0.39 *** (0.175–0.601)	0.31 * (0.01–0.60)	0.10 (−0.14–0.34)	0.19 ** (0.05–0.35)	0.34 ** (0.13–0.56)	0.31 *** (0.141–0.472)
Male	0.06 (−0.024–0.144)	−0.12 ** (−0.21–−0.02)	0.04 (−0.05–0.13)	0.06 (−0.01–0.15)	0.09 * (0.003–0.19)	0.03 −0.04–0.10)
FCI	0.16 *** (0.112–0.199)	0.13 *** (0.09–0.17)	0.09 *** (0.06–0.14)	0.16 *** (0.13–0.19)	0.155 *** (0.122–0.19)	0.15 *** (0.13–0.18)
Asset index	−0.05 * (−0.089–−0.001)	−0.05 * (−0.09–−0.01)	−0.03 * (−0.07–−0.003)	−0.02 (−0.06–0.01)	−0.032 −0.07–0.005)	−0.04 ** −0.07–−0.01)
Child’s weight	0.04 ** (0.009–0.065)	0.04 ** (0.01–0.07)	0.02 (−0.01–0.06)	0.03 ** (0.001–0.06)	0.017 (−0.012–0.05)	0.03 ** (0.012–0.06)
Mother’s weight	0.005 *** (0.002–0.007)	0.004 ** (0.01–0.07)	0.003 ** (0.001–0.006)	0.003 *** (0.002–0.006)	0.003 ** (0.001–0.005)	0.004 *** (0.002–0.006)
Disability	0.40 (0.295–0.496)	0.27 *** (0.16–0.38)	0.09 * (0.01–0.19)	0.18 *** (0.09–0.28)	0.28 *** (0.188–0.381)	0.27 *** (0.19–0.36)
Constant	−5.28 *** (−6.371–−4.182)	−2.99 *** (−4.61–−1.38)	−1.83 ** (−3.03–−0.63)	−3.08 *** −4.34–−1.82)	−3.54 *** (−4.715–−2.374)	−3.61 *** −4.56–−268)

*** *p* value < 0.001, ** *p* value < 0.01, * *p* value < 0.05; age and child’s head circumference were not significant.

**Table 4:** Coefficient estimates from multivariable linear regression models and regression differences in the ASQ z-scores in round one and round two on individual and household characteristics as well as differences in exposure to solid interventions.

Variables	Change in ASQ Domains, Coefficients (95% Confidence Intervals)					
	Communication	Gross Motor	Fine Motor	Personal/Social	Problem Solving	Total Score
Day-care (Yes)	0.007 (−0.437, 0.451)	0.002 (−0.366, 0.371)	−0.28 (−0.753, 0.190)	0.123 (−0.080, 0.326)	0.091 (−0.220, 0.403)	−0.023 (−0.280, 0.233)
Male	0.097 (−0.050, 0.245)	−0.019 (−0.161, 0.121)	0.101 (−0.049, 0.25)	0.072 (−0.069, 0.214)	0.117 ** (−0.013, 0.247)	0.083 (−0.042, 0.207)
FCI	−0.045 *** (−0.121, 0.030)	0.046 * (−0.02, 0.119)	−0.038 *** (−0.110, 0.032)	−0.025 *** (−0.118, 0.067)	−0.047 *** (−0.11, 0.015)	−0.067 *** (−0.14, 0.006)
Asset index	0.048 (−0.029, 0.125)	−0.001 (−0.086, 0.083)	0.029 (−0.059, 0.118)	−0.015 (−0.099, 0.069)	−0.001 ** (−0.077, 0.0757)	0.025 (−0.053, 0.103)
Child’s weight	0.018 (−0.015, 0.052)	−0.001 (−0.042, 0.0401	−0.0002 * (−0.027, 0.027)	−0.006 (−0.027, 0.015)	0.009 (−0.009, 0.027)	0.005 * (0.013, 0.02)
Mother’s weight	−0.02 ** (−0.079, 0.031)	−0.036 (−0.082, 0.009)	−0.019 * (−0.062, 0.023)	−0.0021 (−0.07, 0.030)	−0.016 (−0.058, 0.027)	−0.039 (−0.081, 0.002)
Disability	0.056 *** (−0.117, 0.230)	−0.189 (−0.327, 0.051)	−0.183 (−0.332, 0.033)	−0.083 (−0.247, 0.082)	0.04 ** (−0.125, 0.206)	−0.153 (−0.302, 0.003)
Constant	−1.06 (−3.182, 1.05)	2.249 (0.103, 4.39)	1.96 (0.133, 3.78)	1.117 (−0.622, 2.857)	−0.418 (−2.03, 1.202)	1.8335 (0.330, 3.336)
Observations Number of villages Adjusted R-square Rho	903 146 0.017 0.25	903 146 0.0003 0.29	903 146 0.02 0.59	903 146 0.002 0.28	903 146 0.002 0.32	903 146 0.006 0.86

*** *p* value < 0.001, ** *p* value < 0.01, * *p* value < 0.05.

## Data Availability

Data is currently not available publicly but can be accessed via the co-authors of the study.
